# Echocardiographic Predictors of Worse Outcome After Cardiac Resynchronization Therapy

**DOI:** 10.5935/abc.20150108

**Published:** 2015-12

**Authors:** Eduardo Arrais Rocha, Francisca Tatiana Moreira Pereira, José Sebastião Abreu, José Wellington O. Lima, Marcelo de Paula M. Monteiro, Almino Cavalcante Rocha Neto, Ana Rosa Pinto Quidute, Camilla Viana A. Goés, Carlos Roberto Martins Rodrigues Sobrinho, Maurício Ibrahim Scanavacca

**Affiliations:** 1 Universidade de São Paulo - USP, São Paulo, SP; 2 Universidade Federal do Ceará - UFC, Fortaleza, CE - Brazil; 3 Universidade Estadual do Ceará - UECE, Fortaleza, CE - Brazil

**Keywords:** Heart Failure/ mortality, Echocardiography, Pacemaker, Artificial, Cardiac Resynchronization Therapy, Risk Factors

## Abstract

**Background:**

Cardiac resynchronization therapy (CRT) is the recommended treatment by leading global guidelines. However, 30%-40% of selected patients are non-responders.

**Objective:**

To develop an echocardiographic model to predict cardiac death or transplantation (Tx) 1 year after CRT.

**Method:**

Observational, prospective study, with the inclusion of 116 patients, aged 64.89 ± 11.18 years, 69.8% male, 68,1% in NYHA FC III and 31,9% in FC IV, 71.55% with left bundle-branch block, and median ejection fraction (EF) of 29%. Evaluations were made in the pre-implantation period and 6-12 months after that, and correlated with cardiac mortality/Tx at the end of follow-up. Cox and logistic regression analyses were performed with ROC and Kaplan-Meier curves. The model was internally validated by bootstrapping.

**Results:**

There were 29 (25%) deaths/Tx during follow-up of 34.09 ± 17.9 months. Cardiac mortality/Tx was 16.3%. In the multivariate Cox model, EF < 30%, grade III/IV diastolic dysfunction and grade III mitral regurgitation at 6-12 months were independently related to increased cardiac mortality or Tx, with hazard ratios of 3.1, 4.63 and 7.11, respectively. The area under the ROC curve was 0.78.

**Conclusion:**

EF lower than 30%, severe diastolic dysfunction and severe mitral regurgitation indicate poor prognosis 1 year after CRT. The combination of two of those variables indicate the need for other treatment options.

## Introduction

Scientific evidence places cardiac resynchronization therapy (CRT) as class I in the major guidelines of pacemaking and congestive heart failure (CHF) for patients on optimal clinical treatment, with *New *York Heart Association (NYHA) functional class (FC) II, III or ambulatory IV CHF, ejection fraction (EF) ≤35%, and intraventricular conduction disorder, mainly of the left bundle branch^1,2^.

However, 30%-40% of the patients submitted to CRT might not have a favorable outcome, requiring surgical treatment, with high risks and costs, and no clinical, hemodynamic or survival benefits^3,4^.

Several studies have been aimed at identifying predictors of response to CRT, but with different patterns of definition of response, using mainly comparisons with the end-systolic volume (ESV) and FC improvement^5^. However, patients considered to be responders according to the analysis of ventricular volumes have no correlation with the clinical improvement observed, the walking test or the quality of life. That discrepancy is not a surprise, considering that CRT acts via several hemodynamic and neuro-humoral pathways^6^.

The effects of CRT involve changes in the systolic or diastolic mitral regurgitation grade, diastolic dysfunction grade, systolic function and dyssynchrony. Therefore, a prognostic assessment or response definition using only one variable, such as left ventricular ESV, lacks accuracy.

Thus, multifactorial indices or scores^7^ are required to more accurately identify predictors of better survival and to designate real responders. Those indices should involve a higher number of variables, with greater sensitivity and specificity.

This study aimed at developing an echocardiographic model to predict the risk of cardiac death or transplantation (Tx) after CRT.

## Methods

This is a prospective, observational study, assessing 116 patients with multisite pacemaker implanted at a university-affiliated tertiary hospital. Assessments were performed in the pre-implantation period (1^st^ analysis) and at 1 year after implantation (2^nd^ analysis). Table 1 shows the characteristics of the study population.

**Table 1:** Baseline characteristics of the study population

**Baseline Variables**	**Results**
Total of patients	116
Male sex	69.83%
Age	64.8 ± 11.1
FC (NYHA) III	68.1%
FC (NYHA) IV	31.9%
Beta-blocker use	88.7%
ACEI use	97.4%
High doses of diuretics	31.9%
Number of previous hospitalizations	108
Chagas heart disease	11.2%
Ischemic cardiomyopathy	29.3%
Dilated cardiomyopathy	59.4%
Previous QRS width	160 ms
LBBB	71.50%
EF (Simpson)	29%
Diastolic dysfunction (grade III/ IV)	41.5%
MR (grade II and III)	46%
LVDD	70 mm
Systolic BP	115 ± 17 mmHg
Posterolateral vein	45.4%
Creatinine	1.1 mg/dL

FC: New York Heart Association (NYHA) functional class; ACEI: Angiotensin-converting enzyme inhibitor; LBBB: Left bundle-branch block; EF: Ejection fraction; MR: Mitral regurgitation; LVDD: Left ventricular diastolic diameter; BP: Blood pressure. Pre- and post-CRT QRS width, ejection fraction and LVDD were expressed as medians (non-normal variables). Age was presented as mean and standard deviation.

Most right ventricular electrodes were positioned in the apical region (84%), and a larger distance was kept between the left ventricular and right ventricular electrodes. The models used were St. Jude Medical, Biotronik, Medtronic, and Guidant in 92, 12, 10, and 2 patients, respectively. Two patients received implantations via mini-thoracotomy. The number of patients selected was based on previous studies with similar outcomes.

Drug doses were modified during the follow-up at the discretion of the attending physicians (3 specialists on cardiac stimulation belonging to the same team), responsible for all clinical and surgical procedures. Six echocardiographic variables considered to be of easy acquisition in daily practice and of clinical usefulness were selected for analysis.

### Echocardiographic Parameters

The echocardiographic parameters were analyzed according to the Brazilian^8^ and North-American guidelines for echocardiography^9^. All echocardiographies were performed by 3 experienced echocardiographers, one of whom was responsible for 70% of the examinations. The GE Vivid 7 Ultrasound System (GE Healthcare, Fairfield, CT, USA) with a 3.5-MHz transducer was used. The echocardiographers, who were blinded to the previous clinical and echocardiographic findings of the patients, had experience with that type of patient.

The systolic function was analyzed by using the Simpson method, in the two- and four-chamber two-dimensional mode, followed by the mean. The ventricular diameters were obtained in M mode, according to the guideline recommendations^9^.

The diastolic function was assessed by using mitral flow (at rest and after Valsalva maneuver), tissue Doppler and flow propagation speed on color M-mode. The diastolic dysfunction was classified into four grades as follows: I, mild; II, moderate or pseudonormal; III, accentuated or with restrictive dysfunction; and IV, severe or with irreversible restrictive dysfunction^10^.

The grade of mitral regurgitation was assessed by use of color Doppler, according to the left atrial filling percentage. In mild reflow, that percentage was below 20%; in moderate, between 20% and 40%; percentages greater than those indicated significant reflow. In that practical context, the Coanda effect was interpreted as moderate reflow when restricted to the atrial lateral wall, and accentuated, when extended to the upper pole of the left atrium.

All patients provided written informed consent, and the study was approved by the Ethics Committee of the hospital.

### Statistical Analysis

The Shapiro-Wilk test was used to check the normality of the distribution of the variables. Ejection fraction and left ventricular diastolic diameter (LVDD) did not have a normal distribution.

The categorical variables were presented as frequencies and percentages, and the continuous variables, as means and standard deviations or medians. The categorical variables were compared by using the Mac-Nemar, Stuart-Maxwell or chi-square tests. The Student *t* test was used to compare the distributions of normal, continuous variables, while the Wilcoxon/Mann-Whitney test, to compare continuous variables without normal distribution. The distributions were considered significantly different for p values lower than 0.05.

The univariate relationship between the echocardiographic variables and cardiac death or Tx was assessed by use of Kaplan-Meier survival curve, log-rank test and Cox regression. The continuous variables were assessed by use of Cox regression in the search for a cutoff point.

A Cox multiple regression model was developed in the first year after CRT to assess the independent contribution of each significant echocardiographic variable previously selected in the Cox univariate model. Variables with a p value < 10% were considered potential confounders. Each variable was included in the multivariate model according to hazard descending order, being excluded when p ≥ 5%.

Logistic regression analysis was performed, using hazard^11^ as an independent variable to measure the risk, and cardiac death/Tx as a dependent variable. The accuracy of the models was assessed by using the ROC curve, with sensitivity and specificity. The model was elaborated by dividing the hazard scores into risk categories according to the number of existing variables and classified as low (class A), intermediate (class B) and high risk (class C). To elaborate the scores, the hazard of individual variables was divided by the highest hazard of the model, multiplied by 100 and rounded to the nearest highest algorithm.

To assess the proportional hazards associated with the predictors, Schoenfeld test was used. Bootstraping was used for the internal validation of the model, with confidence interval for the estimated hazard, generating 10,000 random samples of size 60, without replacing the original data set. For each 10,000 artificial samples, the hazards corresponding to each covariable were estimated. The values were ordinated for each covariable, the 95% confidence interval being observed.

Data were assessed by using the Stata/SE software, version 12.1 (StataCorp LP, College Station, TX, USA), and the “R” software (*R Foundation for Statistical Computing*, Vienna, Austria).

## Results

In the 34.09 ± 17.9 months of follow-up, 29 patients died, accounting for a total mortality of 25%. Cardiac mortality/Tx was 16.3%, corresponding to 19 patients, 6 of whom underwent Tx during the study period, 5 due to refractory CHF and 1 due to arrhythmic storm. Of the 6 transplanted patients, 3 died due to advanced disease when undergoing Tx.

In the Cox multivariate model, the variables EF < 30%, diastolic dysfunction and mitral regurgitation were independently related to increased cardiac mortality/Tx, with hazard ratios of 3.1, 4.63 and 7.11, respectively (Table 2).

**Table 2:** Model with the echocardiographic variables in the first year

**Variable**	**Hazard**	**p value**	**95% CI**
MR	7.115132	0.001	2.26449 - 22.35604
Diastolic dysfunction	4.631782	0.004	1.631656 - 13.14824
EF < 30%	3.101647	0.035	1.083580 - 8.878182

CI: Confidence interval; Hazard: Hazard ratio; MR: Mitral regurgitation grade III (severe) as compared to grades II and I (moderate to mild); diastolic dysfunction - grades III and IV (severe) as compared to grades I and II (mild to moderate dysfunction); EF: Ejection fraction.

**Figure 1:**
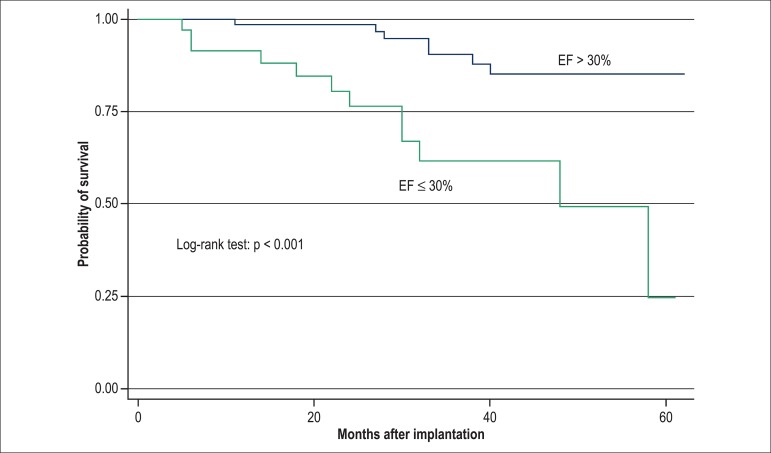
Kaplan-Meier curve for the variable ejection fraction (EF) dichotomized between > 30% and ≤30% at the 2nd phase of the analysis (1 year) exclusively with the echocardiographic variables, p < 0.001, compared by using log-rank test.

**Figure 2:**
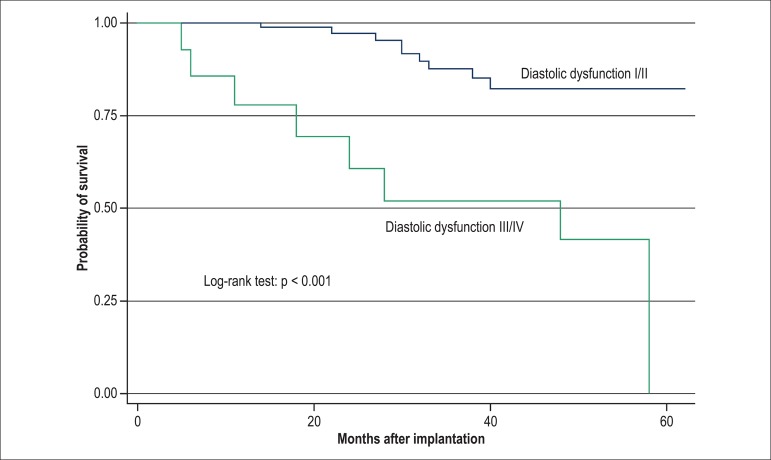
Kaplan-Meier curve for the variable diastolic dysfunction (grades I and II compared to grades III and IV) at the 2nd phase of the analysis (1 year), with p < 0.001, compared by using log-rank test.

**Figure 3:**
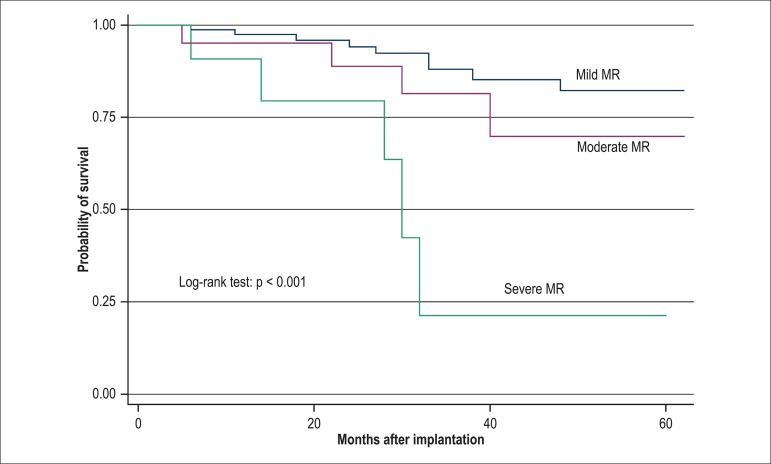
Kaplan-Meier curve for the variable mitral regurgitation (MR), comparing the grades mild, moderate and severe at the 2nd phase of the analysis (1 year), with p > 0.001, by using log-rank test.

The significant variables in the multivariate model were also significant separately in the Kaplan-Meier model, when compared by using log-rank test (p < 0.001) (Figures 1-3). The analysis of the model by using the ROC curve showed an area under the curve (AUC) of 0.785, with sensitivity of 56.2%, specificity of 94.1% and accuracy of 88.2%.

For the models proposed, all variables were assessed for compliance with the proportional hazards assumption by using the Schoenfeld test (Table 3), with results confirming the model adjustment for the variables proposed. The 95% confidence interval was obtained and confirmed the statistical significance of the estimates of the proportions. Thus, the model was validated by bootstraping and showed no lack of adjustment or exaggerated sensitivity of data.

**Table 3:** Proportional hazards test with the echocardiographic variables

**Variable**	**H**	**chi^2^**			**df**			**p value**
MR	-0.01674	0.00			1			0.9456
Diastolic dysfunction	-0.43109	2.52			1			0.1127
EF < 30%	-0.09445	0.14			1			0.7096
Result		2.84			3			0.4174

Shoenfeld test; chi^2^: Chi-square test; p value: Statistical significance level; H: Baseline hazard; df: Degree of freedom; MR: Mitral regurgitation; EF: Ejection fraction.

**Figure 4:**
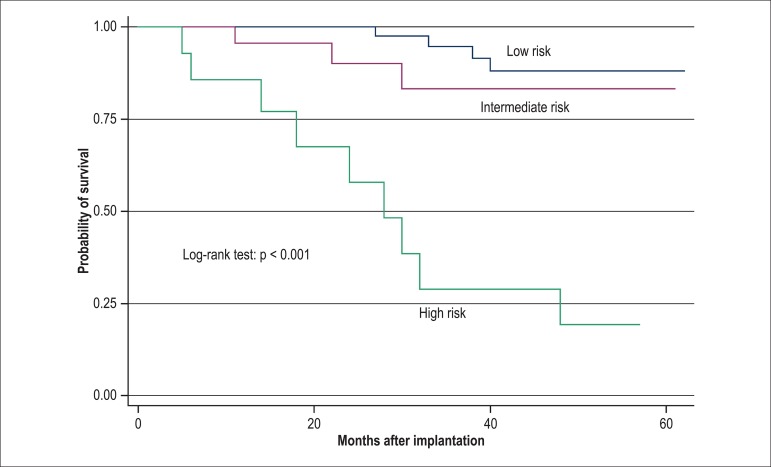
Model with the echocardiographic variables at the first year: Class A - no variable (low risk of cardiac death or transplantation); Class B - presence of 1 variable (intermediate risk); class C (high risk) - presence of 2 or 3 variables (ejection fraction < 30%, grades III and IV diastolic dysfunction compared to grades I and II, and grade III mitral regurgitation compared to grades II and I). Class A implies an event-free rate (EFR) of 97.5% in 30 months, Class B implies an EFR of 83.1%, and Class C, an EFR of 38.5% in 30 months.

From the combination of those variables, we elaborated a model and score with 3 classes as follows: class A (low risk for cardiac death/Tx), corresponding to the absence of the significant variables from multivariate analysis, implying an event-free rate (EFR) of 97.5% in 30 months. The presence of 1 variable (class B) implied an EFR of 83.1% in 30 months, and the combination of 2 or 3 variables (class C) implied an EFR of 38.5% in 30 months (Figure 4 and Table 4).

**Table 4:** Score with the echocardiographic variables in the first year

**Variable**	**Hazard**	**N**	**Score**	**Class**	**Risk**
None	1.0	62	0	A	Low
EF < 30 %	3.1	20	3	B	Intermediate
DD	4.6	3	5	B	Intermediate
MR	7.1	3	7	B	Intermediate
EF + DD	14.3	7	8	C	High
EF + MR	22.0	4	10	C	High
DD + MR	32.9	1	12	C	High
EF + MR + DD	102.2	2	15	C	High

Hazard: Proportional hazards; EF: Ejection fraction; DD: Diastolic dysfunction grades III and IV (severe) compared to grades I and II (mild to moderate); MR: Mitral regurgitation grade III (severe) compared to grades II and I (moderate to mild); Class A - low risk of cardiac death or transplantation; Class B - intermediate risk of cardiac death or transplantation; Class C - high risk of cardiac death or transplantation. Hazard was used as an independent variable in the logistic regression model to elaborate the score. The score was obtained by dividing the variable ‘proportional hazard’ by the highest value, multiplying by 100 and rounding to the nearest number The scores of the combined variables resulted from adding their individual values.

## Discussion

Three important echocardiographic variables (grade III mitral regurgitation^12^, grade III/IV diastolic dysfunction^13^, and EF ≤30%), when present 1 year after CRT, were predictors of cardiac death or Tx in this echocardiographic model.

The model was developed in a high-risk population, which had a median EF of 29%, LVDD of 70 mm, severe diastolic dysfunction in 42% of the sample, moderate or severe mitral regurgitation in 45.6%, and hospitalization due to CHF in the past year in 64%.

The overall mortality rate in this study was 25% (29/116) in 34 ± 17 months, while, Yu et al^5^, in a study including patients with EF of 40% and FC II, therefore at lower risk, have reported an overall mortality rate of 15.6% in 24 months. The CARE-HF study, with a follow-up of 29.4 months, has reported a mortality rate of 30% in the group without intervention as compared to 20% in the group undergoing CRT^14^. The COMPANION study, in a 24-month follow-up, has reported a mortality rate of 21% (131/617) in the group undergoing CRT as compared to 25% (77/308) in the control group^15^. Therefore, the mortality rate in our study is within the range reported in large studies.

In a CARE-HF substudy^16^, severe mitral regurgitation at 3 months was a predictor of total mortality, while in the study by Cabrera-Bueno et al., it was a predictor of worse clinical outcome and smaller reverse remodeling^17^. An Insync ICD substudy has not confirmed those findings for moderate mitral regurgitation^18^. Verheart et al^19^, in a study with 266 patients, have reported earlier significant ventricular remodeling in the group with moderate to severe mitral regurgitation. Regarding our study’s population, 46% had moderate to severe mitral regurgitation before implantation as compared to 28.5% in the first year (p < 0.008 ), showing the beneficial effect of CRT in reducing mitral regurgitation and the high risk of severe mitral regurgitation 1 year after CRT.

Diastolic dysfunction has shown to play a role in CRT, but with no value as an independent predictor of response in most studies. One physiopathological mechanism known to relate to the clinical improvement and better outcome of those patients concerns the reduction in diastolic dysfunction grade^20^. In that study, 41.5% of the patients had severe diastolic dysfunction (grades III and IV), while in the first and second years, only 13.5% and 21.4%, respectively. This can explain the clinical improvement of some patients with no correlation with remodeling or increase in EF^21^. In our study, 41.5% of the patients had grade III/IV diastolic dysfunction, and 13.5% in the follow-up (p < 0.001). That differs from the findings of Salukhe et al^22^, who have reported neither clinical improvement nor remodeling in patients with severe diastolic dysfunction.

An EF ≤30% 1 year after CRT implicated a 3.1-fold increase in the risk of cardiac death/Tx. The MIRACLE study^23^ has reported a total 5.9% increase in EF in 6 months, and the CARE-HF study^24^ has reported a 6.9% increase in 18 months, while, in our study, the median EF was 29% before implantation, 33% 1 year after, and 35% 2 years after implantation. Linde et al^25^, in a REVERSE study subanalysis*,* have shown that a baseline EF < 30%, as compared to values of 30%-40%, related favorably to post-CRT survival, via an index comprising clinical and echocardiographic variables, while Kronborg et al^26^ have shown that a baseline EF < 22.5% increased post-CRT mortality. In a previous study, we observed that a baseline EF < 25% correlated with worse cardiac outcome, which was not maintained after 1 year when assessed with other clinical variables^27^.

In a study^28^ with 65 chagasic patients with implantable automatic defibrillators, on a 40 ± 26.8-month follow-up, we observed annual mortality of 6.1% and no sudden death. In multivariate analysis, an EF < 30% and low educational level were predictors of worse prognosis^28^. The present study had 11.2% of chagasic patients. In another study by our team, Chagas disease was related to an increase in the risk of death only on univariate analysis, probably exceeded by right ventricular dysfunction, a variable not assessed in this study^27^.

The models were properly assessed by using proportionality tests and were internally validated, which increases the value of the data presented.

Patients with at least 2 variables related with worse prognosis 1 year after CRT (grade III mitral regurgitation, grade III/IV diastolic dysfunction and EF ≤30%) should be assessed early in the search for other therapeutic options, such as electrode implantation in another left ventricular position, multisite left ventricular stimulation, mitral valve surgery, earlier cardiac Tx or artificial heart, considering the high cardiac mortality or need for emergency Tx of that population.

### Limitations

This was a single-center study with a small sample and no intra- and inter-observer variability analysis between the echocardiographers. Several important echocardiographic variables were excluded.

## Conclusion

An EF < 30%, severe diastolic dysfunction and severe mitral regurgitation 1 year after CRT indicate worse prognosis, and other therapeutic options should be considered in the presence of 2 of those variables.
